# Species Specificity of Bacteria Associated to the Brown Seaweeds *Lobophora* (Dictyotales, Phaeophyceae) and Their Potential for Induction of Rapid Coral Bleaching in *Acropora muricata*

**DOI:** 10.3389/fmicb.2016.00316

**Published:** 2016-03-21

**Authors:** Christophe Vieira, Aschwin H. Engelen, Linda Guentas, Tânia Aires, Fanny Houlbreque, Julie Gaubert, Ester A. Serrão, Olivier De Clerck, Claude E. Payri

**Affiliations:** ^1^IFD, Sorbonne Universités, UPMC Univ Paris 06Paris, France; ^2^UMR ENTROPIE (UR, IRD, Centre National de la Recherche Scientifique), Institut de Recherche pour le DéveloppementNouméa, New Caledonia; ^3^Phycology Research Group and Center for Molecular Phylogenetics and Evolution, Ghent UniversityGhent, Belgium; ^4^Centre of Marine Sciences, University of the AlgarvePortugal; ^5^Laboratoire MAPIEM EA 4323, Université de ToulonLa Garde, France; ^6^Laboratoire LIVE, Université de Nouvelle-CalédonieNouméa, New Caledonia

**Keywords:** coral bleaching, Illumina sequencing, *in situ* bioassay, *Lobophora*, macroalgal–coral interaction, macroalgal bacterial assemblage, macroalgal culturable epibacteria

## Abstract

While reef degradation is occurring worldwide, it is not uncommon to see phase shifts from coral to macroalgal dominated reefs. Numerous studies have addressed the mechanisms by which macroalgae may outcompete corals and a few recent studies highlighted the putative role of bacteria at the interface between macroalgae and corals. Some studies suggest that macroalgae may act as vectors and/or foster proliferation of microorganisms pathogenic for corals. Using a combination of high throughput sequencing, bacterial culturing, and *in situ* bioassays we question if the adversity of macroalgal-associated bacteria to corals is mediated by specific bacterial taxa. Using Illumina sequencing, we characterized and compared the bacterial community from two *Lobophora* (Dictyotales, Phaeophyceae) species. The two species presented distinctive bacterial communities. Both species shared approximately half of their OTUs, mainly the most abundant bacteria. Species-specific OTUs belong to Planctomycetes, Proteobacteria, and Bacteroidetes. In total, 16 culturable bacterial strain were isolated and identified from the *Lobophora* surface, consisting of 10 genera (from nine families, four classes, and three phyla), some of which are not known as, but are related to pathogens involved in coral diseases, and others are naturally associated to corals. When patches of marine agar with 24 h cultures of each of these bacteria were placed in direct contact with the branches of the scleractinian coral *Acropora muricata*, they caused severe bleaching after 24 h exposure. Results suggest that regardless of taxonomic affinities, increase in density of these bacteria can be adverse to corals. Nevertheless, the microbial community associated to macroalgal surface may not represent a threat to corals, because the specific bacterial screening and control exerted by the alga preventing specific bacterial proliferation.

## Introduction

Competition between benthic macroalgae and corals, two ecosystem engineers of tropical reefs, is a key process shaping the structure of reef communities. Contrary to healthy reefs where macroalgae and corals maintain a stable coexistence, in disturbed reef ecosystems, macroalgae often gain dominance over scleractinian corals. While declines in coral cover are generally associated with increases in the abundance of fleshy (Hughes, 1994; McClanahan et al., 1999), and crustose coralline algae (Antonius and Afonso-Carillo, 2001; Pueschel and Saunders, 2009), in many cases it remains unclear whether the algae are directly or indirectly responsible for coral death or whether they simply settle on dead coral surfaces which are newly open substrate (McCook et al., 2001). In the pursuit of deciphering the mechanisms by which macroalgae may outcompete corals, the first studies focused on effects directly attributable to the alga, e.g., overgrowth, shading, abrasion, recruitment barriers, and allelopathic interactions (see McCook et al., 2001 for review). The concept of holobiont initially proposed for corals (Rohwer et al., 2002) and more recently adopted for algae (Barott et al., 2011) raised the awareness that the microbial component may play a significant ecological role in biotic interactions. As shown for corals (Rohwer et al., 2002; Reshef et al., 2006; Kvennefors et al., 2010; Mouchka et al., 2010), there is increasing evidence suggesting that algal-associated microbiota are species-specific (Sapp et al., 2007; Barott et al., 2011; Hollants et al., 2013a,b) and play an important role in the normal functioning of the algal host (Provasoli and Pintner, 1980; Keshtacher-Liebso et al., 1995; Nakanishi et al., 1996; Matsuo et al., 2003; Croft et al., 2005, 2006; Joint et al., 2007). By extension, the microbiota may also partake important roles in the ecological interactions (e.g., herbivory, competition, etc.) of the algal hosts with other organisms. The microbiota associated to corals and algae feasibly play a role in the outcome of the competition between these major coral reef components. A series of studies indicate (1) that macroalgae can act as reservoirs and vectors of coral pathogens (Nugues et al., 2004; Barott et al., 2011; Sweet et al., 2013) and (2) that macroalgal diffusible compounds can lead to changes in coral microbial assemblages resulting in coral vulnerability or even mortality (Smith et al., 2006; Morrow et al., 2011, 2012). Macroalgae may thus disturb microbial communities of the corals, and convey and/or foster the development of pathogenic bacteria to corals. These results lead to question whether only the known coral-pathogenic bacteria cause bleaching of coral, or if any proliferating bacteria may cause harm to corals.

In coral reef ecosystems the brown algal order Dictyotales (Phaeophyceae) represents one of the most important algal groups, and the genus *Lobophora* (Dictyotales, Phaeophycea) plays a particularly important ecological role in algal–coral–grazing interactions and competition (Coen and Tanner, 1989; Nugues and Bak, 2008; Diaz-Pulido et al., 2009; Anthony et al., 2011; Slattery and Lesser, 2014). *Lobophora* can outcompete coral species (Jompa and McCook, 2002a,b; Nugues and Bak, 2006) and it can produce allelopathic compounds acting against corals (Rasher and Hay, 2010; Slattery and Lesser, 2014; Vieira et al., 2016). Nevertheless, association with live corals is restricted to only specific *Lobophora* species (Vieira et al., 2014, 2016). However, in these studies it is difficult to demonstrate whether the allelochemicals responsible for coral bleaching, are produced by the alga or the associated microbiota. Kubanek et al. (2003) isolated and characterized a complex molecule with antifungal properties from a *Lobophora* species and discussed the possibility that the new compounds could be the product of a microbial symbiont. It therefore seems imperative that future studies need to specifically address the ecological roles that microbial communities associated with macroalgal have.

The present study focuses on *Lobophora*-associated bacteria, characterizes the bacterial community associated to the macroalgae, and assesses the adversity of bacterial isolates of this macroalgal-holobiont on the Scleractinian coral *Acropora muricata* Linnaeus (1758).

## Materials and methods

### Study area and collection of samples

**Figure 1:**
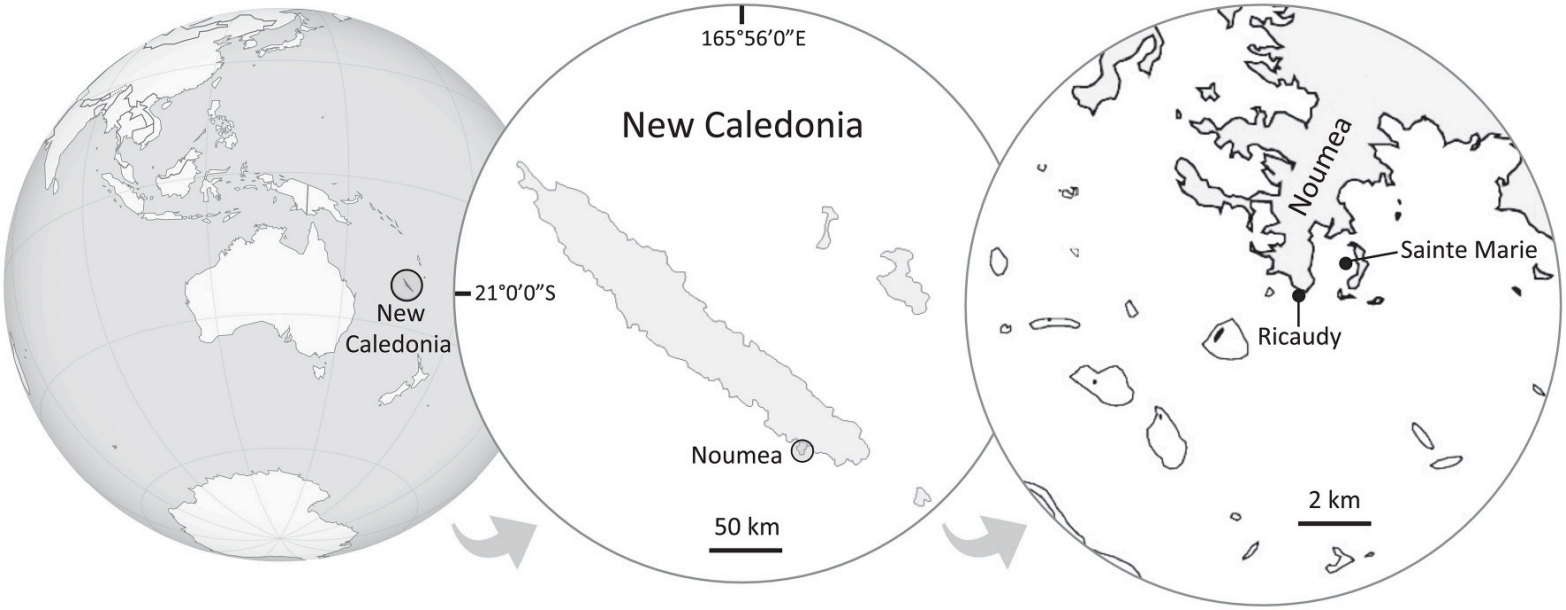
**Map of New Caledonia showing the sampling and bioassays sites**. *Lobophora rosacea* was collected in Ricaudy and *L. monticola* in Sainte Marie Bay.The distance between the two sites is ~1.2 km.

**Figure 2:**
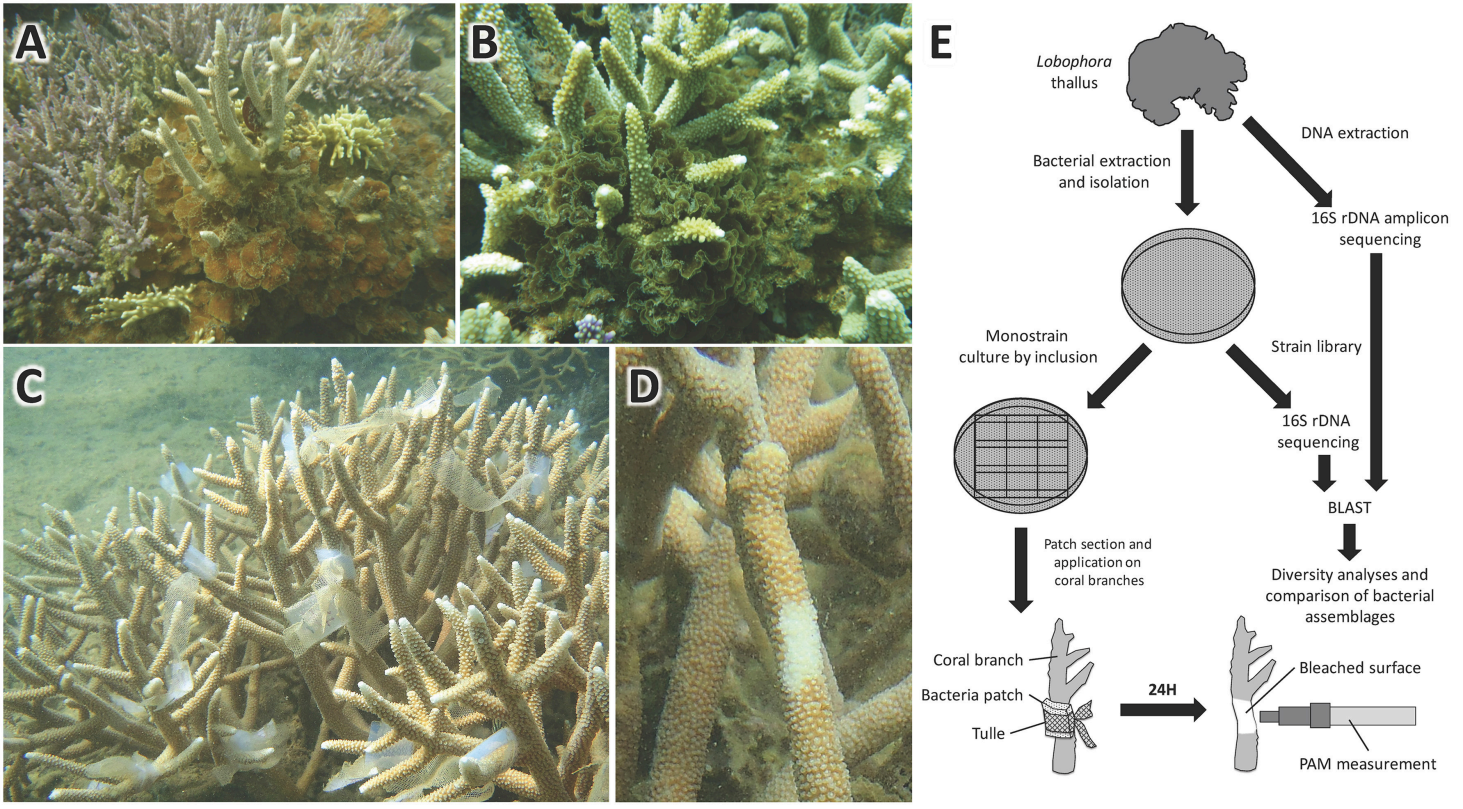
**Pictures of (A) *L. rosacea* and (B) *L. monticola* growing on *Acropora* colonies**. Pictures of *in situ* bioassays on *Acropora muricata* colonies, showing **(C)** the marine agar patches with mono-specific bacterial cultures applied onto *A. muricata* colonies branches, and **(D)** the bleaching induced by the bacteria after 24 h exposure. Flowchart of the method deployed from the bacterial extraction on the algal-surface to the bioassays and bacterial diversity characterization **(E)**.

This study was performed in Noumea lagoon, New Caledonia, where two species of *Lobophora* were collected in two different sites, both fringing reefs, located 1.2 km apart (Figure 1). *Lobophora monticola* was collected in Sainte Marie Bay at 5 m depth (latitude: −22.297713°, longitude: 166.481639°) and *Lobophora rosacea* in Ricaudy at 2 m depth (latitude: −22.315317°, longitude: 166.457717°; Figure 1). The two species are naturally found growing associated with corals. *L. monticola* is generally found growing attached to *Acropora* corals, and *L. rosacea* is commonly found growing niched within branching corals such as *Acropora*. The site in Sainte Marie Bay is located several meters away from the mangrove shore, and is protected from wind exposure, resulting in turbid and still waters. The site in Ricaudy is exposed to dominant wind and thus experiences strong wave action. Algal thalli were collected right off their coral substrates (i.e., *Acropora*, Figures 2A,B); epiphyte-free (i.e., algal epiphytes) specimens were specifically selected. For Illumina sequencing, specimens were put in ziploc bags under water, stored in a cooler, frozen at −80°C, and freeze-dried. For the bacterial isolation and culture, algal thalli were rinsed three times consecutively with sterile seawater right after collection on the boat, placed in sterile vials, and stored in a cooler. Once in the laboratory, thalli were rinsed one more time with a sterile saline solution (36 g.L^−1^) under a biological safety cabinet.

### Bacterial community barcoding and characterization

#### Bacterial DNA extraction and amplification

DNA was extracted from five freeze-dried replicates (a replicate = a unique specimen) of each species (*L. rosaceae* and *L. monticola*) using the Quick-gDNA kit (Zymo Research™) applying the manufacturer protocol.

The total 16S rDNA region was amplified using the universal primers 27F and 1494R (Lane, 1991) with the following changes to the original protocol: after an initial denaturation at 95°C for 2 min, conditions were 35 cycles of denaturation at 95°C for 20 s, annealing at 55°C for 20 s, and extension at 72°C for 90 s. The final extension was at 72°C for 3 min. The 25 μl reaction mixture contained 250 μM dNTPs, 0.6 μM of each primer, 1 × 2PCR buffer mix, 2 μl of template DNA (with a final concentration of about 10 ng μl–1), and 0.3 μl of Taq polymerase (Advantage® 2 Clontech). PCR products were cleaned using ExoFastAP enzyme following the manufacturer protocol (Thermo Scientific™) and amplified DNA was submitted to Molecular Research (MR DNA), Shallowater, Texas where a nested-PCR was performed prior to sequencing. Modified 8 bp key-tagged primer 799F along with the reverse primer 1193R (fragment ~400 bp), which avoid chloroplast cross amplification (Bodenhausen et al., 2013), were used and PCR conditions were as follow: 95°C for 3 min, 30 cycles of 95°C for 20 s, 50°C for 30 s, 72°C for 30 s, and a final elongation of 72°C for 3 min. Samples were pooled together in equal proportions based on their molecular weight and DNA concentrations and purified using calibrated Ampure XP beads. DNA libraries were prepared by using a Illumina TruSeq DNA library preparation protocol and paired-end (2 × 250 bp) sequencing performed at MR DNA (www.mrdnalab.com, Shallowater, TX, USA) on a MiSeq following the manufacturer's guidelines.

#### 16S rRNA analysis and bacterial community diversity

All the diversity analyses were performed using the program QIIME 1.8.0: Quantitative Insights Into Microbial Ecology (Caporaso et al., 2010). Sequences were screened for a minimum read length of 350 bp and < 2 or more undetermined nucleotides. The filtered dataset, containing only high quality sequences, was submitted to a conservative chimera detection filter using the ChimeraSlayer method (Haas et al., 2011). Selected high quality chimera-free sequences were clustered into Operational Taxonomic Units (OTUs) using the UCLUST (Edgar, 2010) with a pairwise identity threshold of 0.97.

Representative sequences for each OTU were picked using the “most-abundant” method and OTU sequence alignment was performed with Pynast (Caporaso et al., 2010). The Ribosomal Database Project (RDP; Wang et al., 2007) classifier was used for taxonomic assignment with a 95% confidence threshold. Sequences with the best match for eukaryotes (i.e., chloroplasts and mitochondria) were excluded from the OTU table in downstream analyses. To assign each OTU to the closest matching described taxon, searches were performed against the Greengenes reference database (version 12_10; McDonald et al., 2012) with a maximum *e*-value to record an assignment of 0.001. The degree of relatedness of the subsets of the most common sequences was inferred using the phylogenetic reconstruction with Qiime's script make_phylogeny.py and using, by default, FastTree (Price et al., 2010) from Qiime.

#### Diversity estimation and comparison of bacterial assemblages

In order to obtain direct descriptors of the diversity of bacterial assemblages, we calculated two widely used diversity indices, the Shannon (*H*′) and the Simpson indices of diversity (1 – *D*). The non-parametric ACE and the Chao 1 richness index were calculated with the software estimates (Version 9; Colwell, 2013) to estimate microbial diversity. Principal component and cluster analyses were used to determine the similarity between the samples and the species. To compare the difference of community composition between the two *Lobophora* species an analysis of similarities (ANOSIM) was performed in R (R Development Core Team, 2013) using the “vegan” package. Finally, to identify which bacterial groups mostly contribute to the difference between the two *Lobophora* species, a Similarity Percentages (SIMPER) analysis was performed in R using the “vegan” package.

### Bacterial culture and bioassays

#### Bacterial extraction

Bacteria were extracted from the plant surface by implementing three independent extraction methods on different thalli. For the first method, the bacteria were extracted by swapping the thallus surface with a cotton swab and streaking it on a marine agar (MA, Laboratorios Conda) plate. Then the swab cotton was introduced into a sterile Falcon tube with 10 mL of Marine Broth (MB, Laboratorios Conda) and shaken vigorously for 1 min and incubated 48 h at 30°C, under shaking conditions (120 rpm). The second method consists of inoculating a MA petri dish by directly placing an algal thallus on it. After firmly pressing it against the agar, the thallus was left 5 min on it. The process was renewed with the other side of the thallus on the same petri dish and the plates were incubated at 30°C during 48 h. In the third method, a thallus was placed into a 15 mL sterile tube with 10 mL of sterile saline water and shaken for 1 min. Three MA plates were inoculated by spreading, respectively, 100 μL of the 1/10, 1/100 dilutions and pure suspension. Plates were then incubated 48 h at 30°C.

#### Bacterial isolation

After the 48 h incubation, bacteria presenting visually diverse colony morphologies were selected for isolation. A small portion of a bacterial colony was gently scooped with a disposable sterile inoculating loop and streaked into a MA plate, which was then incubated 48 h at 30°C. This process was renewed two more times to assure pure isolates. Several colonies of a given bacteria were then placed into 10 mL of MB within a 50 mL sterile tube and incubated 24 h at 30°C. Hundred microliters of the resulting culture was spread onto a MA plate and incubated 24 h at 30°C. Finally, colonies picked from the marine agar plates and introduced in 800 μL of MB or 800 μL of bacteria from the liquid culture were put together with 800 μL sterile 25% glycerol solution into a cryotube and stored at −80°C.

#### Bacterial DNA extraction and identification

Bacterial DNA was extracted with the DNeasy® Blood and Tissue Kit (Qiagen, California, USA), following the manufacturer's instructions. The 16S rRNA gene (1500 pb) was used for bacterial identification. Amplification of the 16S rRNA gene by PCR, using the universal 16S rRNA primers (Table S1), was followed by sequencing of the resulting PCR amplicons. Identification to the species level was performed by comparison to a Basic Local Alignment Search Tools (BLAST) database comprising over 1 million entries of bacteria.

#### *In situ* bioassays

A flowchart illustrating the methodology is given in Figure 2E. Isolated bacteria were put in direct contact with the coral branches by means of marine agar patches. The marine agar was inoculated by inclusion in order to have the bacteria growing on and within the marine agar. To do so, 100 μL of bacteria (concentration of ca. 30 bacteria/mL) from the marine broth culture were put directly on the empty petri dish after which the marine agar, which had cooled down to room temperature, was poured on the petri dish. The mix, marine agar with the 100 μL bacteria solution, was then gently mixed to homogenize the bacteria into the marine agar. The plates were then incubated 24 h at 30°C. Ten agar strips of 2 cm^2^ were cut from the petri dish under the biosafety cabinet and put into sterile re-sealable zipper storage bags and stored into a cooler until application. The agar strips were then applied on the coral branches and fixed to it with sterile labeled tulle bands (Figures 2C–E). Seawater bacteria were used as control in addition to sterile marine agar patches. After 24 h application on the coral branches, the agar strips were removed. Bioassays were performed *in situ* directly on coral colonies. A total of 10 replicas were used, with one coral colony representing one replica, on which the 16 isolated strains were tested separately, resulting in a total of 160 bioassays.

#### Coral bleaching measurement and statistical analyses

Pulse Amplitude Modulated (PAM) fluorometry was used to assess the effects of bacteria on coral health (effective quantum yield). PAM fluorometry measurements were carried out with a Diving-PAM (Walz) directly after removal of the strips. PAM fluorometry measures the photosynthetic efficiency of photosystem II within the endosymbiotic *Symbiodinium* spp. that may be used as a quantitative measure of photoinactivation during coral bleaching (Warner et al., 1999). PAM fluorometry values of healthy corals range between 0.5 and 0.7, depending on the coral species and time of the day. Values between 0 and 0.2 are indicative of severe bleaching or mortality (Fitt et al., 2001). PAM fluorometry measurements were made right where the strips were applied and 5 cm next to it, as a spatial control in order to have a coral health baseline for comparison. Data were tested for normality with a Shapiro–Wilk test. Since the data respected the parametric assumptions, a one-way ANOVA was subsequently performed, followed by the Tukey *post-hoc* honestly significant difference (HSD) test. Statistical analyses were performed using R.

## Results

### Bacterial community characterization through 16S rRNA NGS and culturing

#### Characterization through 16S rRNA NGS

**Figure 3:**
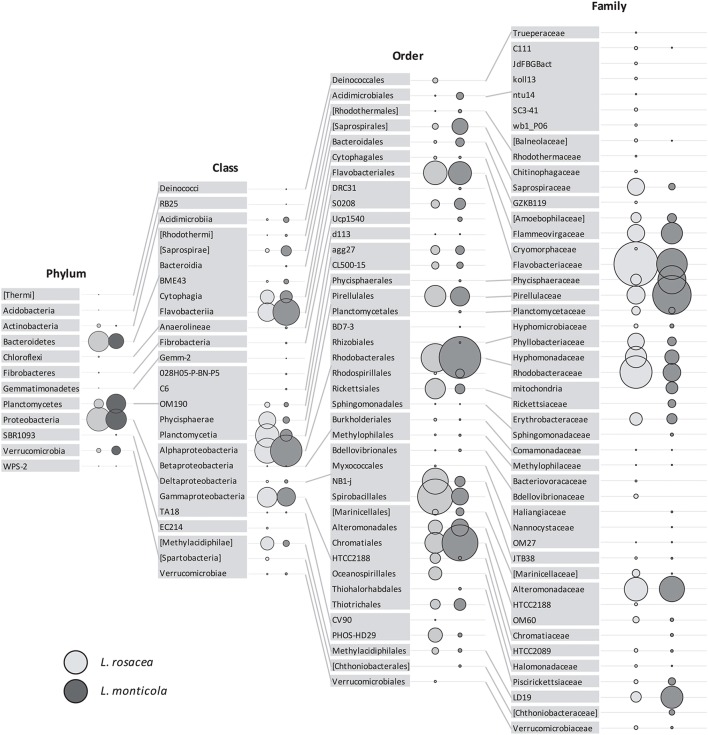
**Relative abundance of the bacterial taxa, present in at least four replicates per species, associated with *Lobophora rosacea* (light gray bubbles on the left side) and *L. monticola* (dark gray bubbles on the right side), at different taxonomic levels (Phylum, Class, Order, Family)**.

In a dataset of more than 644,377 sequences obtained from the 10 *Lobophora* specimens studied, 3247 and 3313 OTUs were identified from *L. rosacea* and *L. monticola*, respectively. On average each sample had 1386 ± 162 OTUs (Table S2). Shannon and Simpson diversity indices were calculated for each sample and were used to compare the gross structure of bacterial assemblages among our samples. Both indices were rather stable across replicate samples, with an average value of 5.14 ± 0.32 and 5.67 ± 0.19 (*H*′), and 0.977 ± 0.010 and 0.989 ± 0.003 (1 – *D*), for *L. monticola* and *L. rosacea*, respectively. The Shannon (*F* = 10.0372, *p* = 0.1675) and Simpson (*F* = 6.2934, *p* = 0.2321) indices were not statistically significant different between the two species. The non-parametric estimators predicted an average number per sample of 2777 ± 606 and 2839 ± 386 OTUs (ACE), and of 2805 ± 646 and 2881 ± 604 (Chao1) OTUs for *L. monticola* and *L. rosacea*, respectively. Chao1 (*F* = 0.0374, *p* = 0.9857) and ACE (*F* = 0.0269, *p* = 0.9897) both richness estimators were not statistically significant different between the two species. Comparison of the core OTUs shared by at least four replicas per species between the two *Lobophora* species is presented in Figure 3. The sequences are publicly available in MG-RAST with the accession numbers 467520195.3–4675204.3.

#### Comparison between *Lobophora* species bacterial assemblages

**Figure 4:**
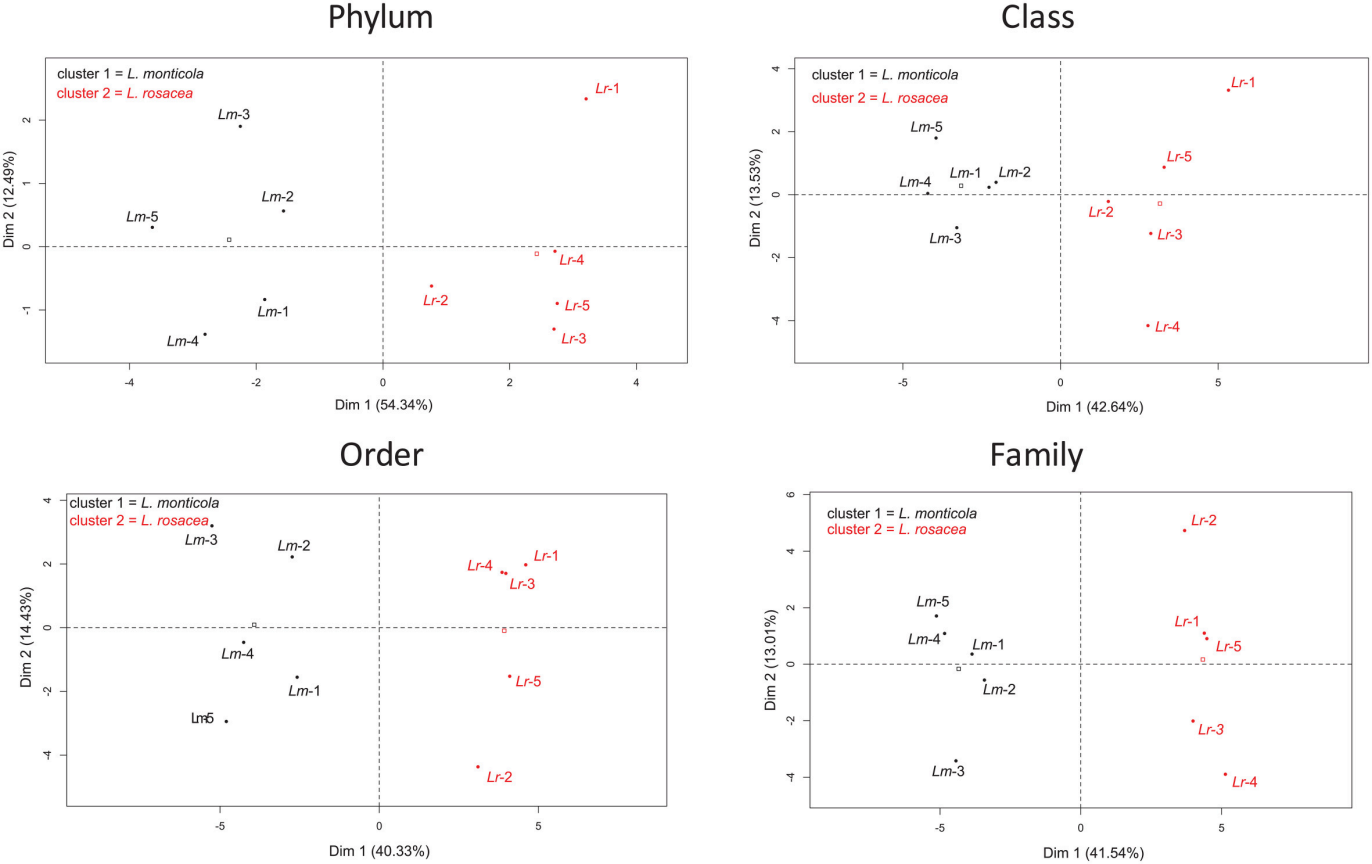
**Principal Component Analysis (PCA) for the bacterial assemblages from 10 samples of two *Lobophora* species, *L. monticola* (*Lm-x*), and *L. rosacea* (*Lr-x*)**.

The ANOSIM analysis showed a statistically significant difference in the community composition at all taxonomic levels (*R* = 0.488−0.94; *p* = 0.006−0.012) between the two *Lobophora* species. The most influential taxa, determined with the SIMPER analysis, are given in Table S3. We performed PCA analysis to determine if the bacterial assemblage associated to the *Lobophora* species is species-specific. The four analyses resulted in the classification of the ten samples into two distinct clusters corresponding to the two *Lobophora* species. The first component that contributed significantly to explaining the relationships among the samples (eigen-values > 1), by clearly disjointing the two species, accounted for 40–54% of the variance depending on the taxonomic level, with a stronger value at the phylum level (54%; Figure 4). The most abundant phyla were shared by both species (Proteobacteria, Bacteroidetes, Planctomycetes, and Verrucomicrobia).

#### Characterization through culturing

**Figure 5:**
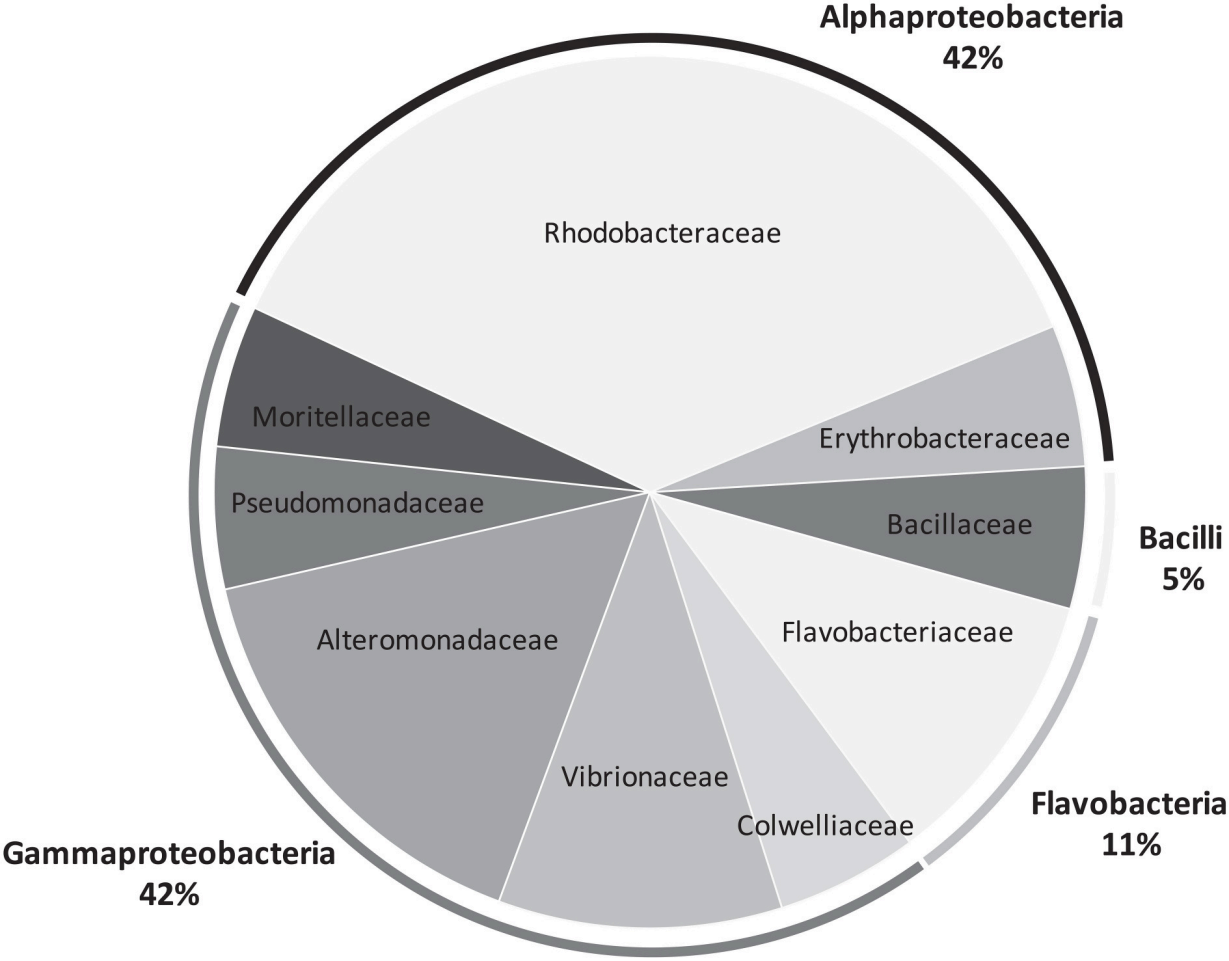
**Pie chart representing the bacterial diversity, at the family and phylum levels, recovered from *Lobophora* surface using culture approaches**.

Bacteria grew quite well after 48 h of incubation, as visually assessed. We managed to culture 20 strains, 10 for each species of *Lobophora*. BLAST-search revealed 16 different strains (Table 1) with similarity to GenBank sequences ranging from 95 to 100% (Table S4). The two species of *Lobophora* were composed of similar percentages of surface-associated cultivable bacteria per phylum, with 85 and 90% Proteobacteria for *L. rosacea and L. monticola*, respectively, and 10% Bacterioides for both species, with an additional 5% *Firmicutes* for *L. rosacea* (Table 1). The isolated and successfully cultured 16 strains belonged to ten different genera: *Bacillus, Erythrobacter, Microbulbifer, Muricauda, Paramoritella, Ruegeria, Shimia, Tenacibaculum, Thalassomonas*, and *Vibrio* (Figure 5). Out of the nine families isolated, three (*Colwelliaceae, Bacillaceae, Moritellaceae*) were not detected and four of these families were abundantly characterized in the Illumina 16S rRNA amplicon sequencing (Alteromonadacea, Rhodobacteracea, Flavobacteriaceae, and Erythrobacteriaceae).

**Table 1:** **List of the bacterial strains, with their GenBank accession numbers, isolated from *Lobophora rosacea* and *L. monticola* using culture approaches, identified with the BLAST-search**.

**Algal host**	**Strain**	**Isolated bacterial species**	**Family**	**Phylum**	**Accession number**
*L. monticola*	LMB	*Ruegeria* sp.2	Rhodobacteraceae	α-Proteobacteria	KU560505
	LMC	*Thalassomonas* sp.	Colwelliaceae	γ-Proteobacteria	KU560494
	LMD	*Ruegeria* sp.3	Rhodobacteraceae	α-Proteobacteria	KU560501
	LME	*Ruegeria* sp.1	Rhodobacteraceae	α-Proteobacteria	KU560502
	LMF	*Vibrio* sp.2	Vibrionaceae	γ-Proteobacteria	KU560496
	LMG	*Microbulbifer* sp.1	Alteromonadaceae	γ-Proteobacteria	KU560493
	LMH	*Tenacibaculum* sp.	Flavobacteriaceae	Bacteroidetes	KU560487
	LMI	*Ruegeria* sp.2	Rhodobacteraceae	α-Proteobacteria	KU560506
	LMJ	*Paramoritella* sp.	Moritellaceae	γ-Proteobacteria	KU560495
	LMM	*Ruegeria* sp.4	Rhodobacteraceae	α-Proteobacteria	KU560504
*L. rosacea*	LR1	*Shimia* sp.1	Rhodobacteraceae	α-Proteobacteria	KU560499
	LR11	*Shimia* sp.1	Rhodobacteraceae	α-Proteobacteria	KU560500
	LR2	*Erythrobacter* sp.	Sphingonmonadaceae	α-Proteobacteria	KU560498
	LR3	*Muricauda* sp.	Flavobacteriaceae	Bacteroidetes	KU560488
	LR4	*Ruegeria* sp.1	Rhodobacteraceae	α-Proteobacteria	KU560503
	LR5	*Vibrio* sp.1	Vibrionaceae	γ-Proteobacteria	KU560497
	LR6	*Microbulbifer* sp.1	Alteromonadaceae	γ-Proteobacteria	KU560492
	LR7	*Microbulbife*r sp.2	Alteromonadaceae	γ-Proteobacteria	KU560491
	LR8	*Bacillus* sp.	Bacillaceae	Firmicutes	KU560489
	LR9	*Microbulbifer* sp.3	Alteromonadaceae	γ-Proteobacteria	KU560490

### *In situ* bioassays

**Figure 6:**
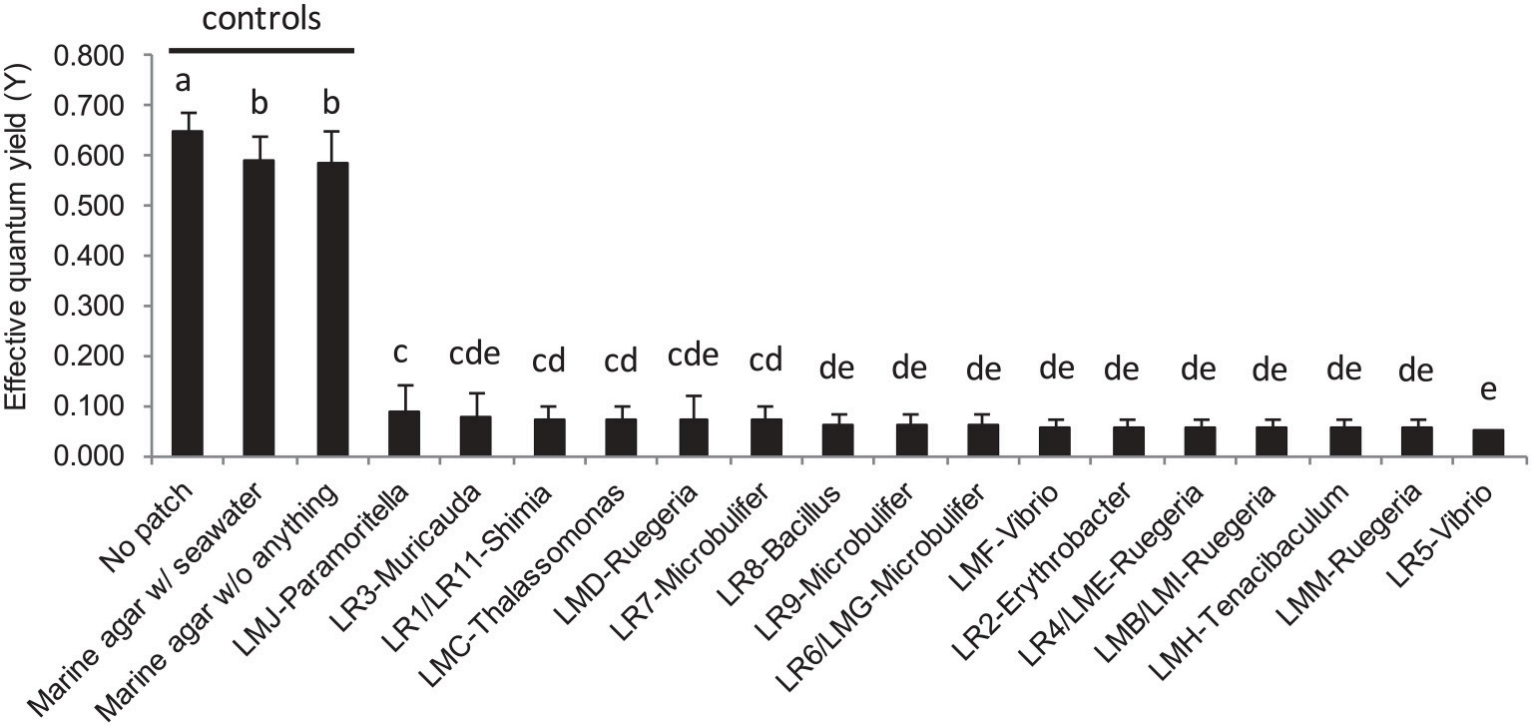
**Barplot representation of the photosynthetic efficiency from *Acropora muricata* measured by Pulse Amplitude Modulated (PAM) fluorometry following the *in situ* bioassays with mono-specific bacterial inclusion culture with the 16 strains isolated from *L. rosacea* and *L. monticola***. The statistical analyses, comparing the bacterial culture patches to control patches, were performed using one-way ANOVA and Tukey's HSD *post-hoc* test. Letters indicate distinct groupings based on *post-hoc* statistical comparison among sub-fractions. *n* = 10 assays. Error bars represent standard deviation of the mean.

After 24 h exposure, the surface area of the coral *A. muricata* in direct contact with each of the macroalgae-associated culturable bacterial patches, showed severe visual bleaching (Figure 2C) and an almost complete suppression of coral photosynthetic efficiency across all tested strains, with a relative average quantum yield decrease to 0.064 ± 0.051 (± *S.D.*), (*p* < 0.001; Figure 6). Nevertheless, coral tissue on which agar patches were applied was left intact. Seawater bacterial patches, used as control in addition to “sterile” marine agar patches, did not cause coral bleaching.

## Discussion

This study shows that all culturable bacteria isolated from macroalgae of the genus *Lobophora*, pertaining to various taxa, caused severe bleaching, and significantly suppressed photosynthetic efficiency of the coral *A. muricata*. Our experiments show that macroalgae can indirectly cause coral mortality by means of their surface-associated microbiota. These results suggest also that any bacterial biofilms might be capable of bleaching corals but, as discussed hereafter, a set of ecological factors are generally preventing specific bacterial growth.

### Bacterial assemblage diversity

This is the first bacterial community characterization of species from the genus *Lobophora* using Illumina metabarcoding sequencing. Our 16S rRNA comparative analyses of the bacterial communities between the two *Lobophora* species showed that the two diversity indices, one more sensitive to richness (Shannon) and the other to evenness (Simpson) were not significantly different between the two species. In contrast, multivariate analyses conducted on Illumina sequencing results clearly showed the species-specificity of the bacterial assemblages associated to individuals of *L. rosacea* and *L. monticola*. The two species mainly differ in the most abundant bacterial taxa among those shared by both species. Results indicate that the two *Lobophora* species have comparable richness of bacterial assemblages but contrasting composition. The two *Lobophora* species grow in habitats with contrasting environmental conditions. Considering the habitat specificity of these two species, it is questionable whether the bacterial assemblage species-specificity could be linked to the different niches that the two species inhabit. Transplant or common garden experiments will allow concluding whether/to what extent *Lobophora* species bacterial community composition is controlled by ecological factors or the evolutionary history of the host.

Similar phyla were found with relatively similar percentages between the two *Lobophora* species. They also shared four bacterial families (i.e., Flavobacteriaceae, Rhodobacteraceae, Alteromonadaceae, Vibrionaceae), three genera (i.e., *Ruegeria, Microbulbifer, Vibrio*), and two OTUs (*Microbulbifer* sp. and *Ruegeria* sp.1). Barott et al. (2011) performed pyrosequencing on the microbial communities of the major ecological functional algal groups in Curaçao (The Netherlands Antilles) including the genus *Dictyota* (Dictyotales, Phaeophyceae), and established a microbial fingerprint for each group. When comparing their results with ours it is clear that our *Lobophora* fingerprints (relative abundance percentage) do not resemble any of the algal groups studied by Barott et al. (2011; Figure S1). To what extent technical (marker), region (Caribbean vs. Pacific) or host specificity play a role is uncertain. When considering the major phyla, the two *Lobophora* species had three major phyla in common with the other algal groups (*Proteobacteria, Bacteoidetes*, and *Planctomycetes*), but did not present Firmicutes. Furthermore, *Lobophora* presented one additional major phylum (*Verrucomicrobia*) not detected in the other algal groups.

### Potential coral pathogens?

The present bioassay results evoke symptoms of “white” diseases, such as the white plague syndrome affecting massive and encrusting corals, the white band disease affecting *Acropora* spp., and the Acroporid white syndrome affecting *A. hyacinthus*. While none of the isolated bacterial OTUs have yet been reported in the literature as coral pathogens the majority of the isolated genera (*Vibrio, Ruegeria, Thalassomonas, Shimia, Microbulbifer*) (Table 1) were documented either as pathogens of corals or other organisms, or associated to coral diseases. Several species of the genus *Vibrio* are known as agents responsible for coral diseases such as the “yellow blotch/band disease” (Cervino et al., 2004; Rosenberg et al., 2007; Bourne et al., 2008, 2009; Sunagawa et al., 2009). Some members of the genus *Thalassomonas* were reported as the causative agent of the “white plague” (Rosenberg et al., 2007). Species of the genus *Ruegeria* presented antibacterial properties (Porsby et al., 2008) and were found associated to the “yellow band disease” (Apprill et al., 2013). The genus *Shimia* was also found associated to corals affected with the “*Porites* White Patch Syndrome” (Séré et al., 2013). Finally the genera *Microbulbifer* and *Bacillus* presented antimicrobial activities (Kim et al., 2008; Nithyanand and Pandian, 2009). When looking at the list of coral-associated bacteria provided by Mouchka et al. (2010) established at the ordinal level, we notice that five of the orders that are more prevalent in infected or bleached corals, were present in *Lobophora*, two relatively abundant orders, Rhodobacterales (8 and 23% for *L. rosacea* and *L. monticola*, respectively) and Alteromonadales (10 and 9%), and three rare orders (Chromatiales, Clostridiales, and Vibrionales). Similarly, Barott et al. (2011) showed that two *Dictyota* species harbored the highest percentages (39.6 and 40.8%) of potential pathogens associated with coral diseases, which supports the idea that members of the Dictyotales hold a reservoir of potential coral pathogens.

### Ecological insight

It is only recently that the microbial component has been considered in the interactions between algae and corals, and we have only begun to scratch the surface of understanding the complex interactions between coral, algae, and microbes (Table 2). Recent studies showed that macroalgae may alter microbial communities of corals, and convey and/or foster the development of pathogenic bacteria to corals (Table 2).

**Table 2:** **Comparison of studies on microbial mediation in macroalgal–coral interaction**.

**Reference**	**Objectives and methods**	**Results and conclusion**	**Algae**	**Corals**
Barott et al., 2011	Microbial diversity analysis; 16S rDNA tag pyrosequencing	Algae serve as reservoirs for potential coral pathogens	CCA, *Dictyota bartayresiana, Halimeda opuntia*, Turf	*Montastra annularis*
Barott et al., 2012	Microbial diversity analysis; 16S rDNA tag pyrosequencing	Algae caused hypoxia on adjacent coral tissue and shifts in the bacterial communities at the interaction zones	CCA, *Dictyota bartayresiana, Halimeda opuntia*, Turf	*Montastra annularis*
Barott and Rohwer, 2012, a review	DAM [dissolved organic matter (DOM), direct contact, disease, algae and microbes] model	Macroalgae promote heterotrophic microbial overgrowth of coral	–	–
Morrow et al., 2011	Effects of allelochemicals from macroalgae and cyanobacteria on coral microorganisms; bacterial bioassays and 16S rDNA sequencing	Alter coral microbiome	*Acanthophora spicifera, Lobophora variegata, Dictyota* sp., *D. pulchella, Lyngbya polychroa, L. majuscula*	*Montastraea faveolata, Porites astreoides*
Morrow et al., 2012	Effects of algal extracts on coral bacterial assemblage; 16S rRNA DGGE	Algal extracts induce bacterial assemblage shifts	*Dictyota* sp., *Halimeda opuntia, Lobophora variegata*	*Montastraea faveolata, Porites astreoides*
Morrow et al., 2013	Effects of algal contact on coral bacterial assemblage; 16S rRNA DGGE	Algal contact induce bacterial assemblage shifts	*Dictyota menstrualis, Halimeda opuntia*	*Montastraea faveolata, Porites astreoides*
Nugues et al., 2004	Effects of algal contact on coral; field experiment	Transmission of coral disease	*Halimeda opuntia*	*Montastraea faveolata*
Smith et al., 2006	Effects of dissolved compounds from algae on corals; laboratory experiments	Dissolved compounds from algae	*Caulerpa*, CCA, *Cyanobacteria, Dictyosphaeria cavernosa, Halimeda, Microdictyon, Peysonnellia*, Turf mixed	*Acropora, Favia, Fungia, Hydnophora, Montastrea, Montipora, Pavona, Pocillopora verrucosa, Porites, Stylophora*
Sweet et al., 2013	Original source of coral pathogens	Algae serve as reservoirs for a variety of different potential coral pathogens. Algal-associated microbes alone are unlikely to cause coral death	*Caulerpa cupressoides, C. racemosa, Chlorodesmis fastigiata, Dictyota frabilis, Halimeda macroloba, Hincksia* sp., *Hydroclathrus clathrus, Hypnea* sp., *Laurencia* sp., *Padina australis, Sargassum polycystum*	*Acropora muricata, Montastraea faveolata*
Thurber et al., 2012	Effects of macroalgae on coral growth and microbial community structure	Algae caused the disappearance of a γ-proteobacterium; increases or decreases in microbial taxa already present in corals; establishment of new taxa to the coral microbiome; vectoring and growth of microbial taxa from the macroalgae to the coral	*Dictyota menstrualis, Galaxuara obtusata, Halimeda tuna, Lobophora variegata, Sargassum polyceratium*	*Porites astreoides*
This study	Effects of macroalgae-associated bacteria on corals; bacterial bioassays and 16S rDNA Illumina sequencing	Macroalgae-associated bacteria induce quick and strong coral bleaching	*Lobophora monticola, L. rosacea*	*Acropora muricata*
Vermeij et al., 2009	Effects of macroalgae and microbes on survival and settlement success of coral planulae	Macroalgae indirectly cause planular mortality by enhancing microbial concentrations or by weakening the coral's resistance to microbial infections	*Ulva fasciata, Acanthophora spicifera, Pterocladiella caerulescens, Sargassum polyphyllum*	*Montipora capitata*

Our findings suggest that (1) *Lobophora* hosts surface and core bacterial orders present in diseased and bleached corals, and epibacteria experimentally capable of bleaching corals, and that (2) all tested bacteria can equally induce strong and quick coral bleaching. The bleaching potential from the tested bacteria is apparently not restricted to bacteria from the genus *Lobophora* considering the panel of bacteria isolated which belong to a variety of taxa but also the natural presence of some of these taxa in healthy corals. In other words, regardless of their taxonomic affinity, dense mature bacterial films can intrinsically bleach corals. But while these results seem to concur with recent findings on the role of macroalgae as conveyors and fosterers of coral pathogens, this does not imply necessarily that macroalgae represent a threat to corals. The mere presence of bacteria is not a threat, as the consequences depend on conditions allowing them to proliferate. In fact, corals themselves also naturally host pathogenic bacteria that can be detrimental to the health of the host under certain conditions. Nonetheless, only specific bacteria have actually demonstrated the capacity to efficiently proliferate and bleach corals. Smith et al. (2006) previously showed that macroalgal diffusible compounds enhanced the activity of coral- or seawater-associated bacteria, leading to coral mortality. These latter results support the idea that bacterial proliferation can generally be adverse to coral. Consequently, although it is true that macroalgae may harbor coral pathogens (Nugues et al., 2004; Barott et al., 2011; Sweet et al., 2013), bacterial adversity is not restricted to the pathogenic strains, but appears related to bacterial density. The natural presence of potentially pathogenic species within coral microbial communities (Mouchka et al., 2010; Barott et al., 2011) supports the idea that adversity toward corals is linked to microbial density. This implies that these specific pathogenic strains have not evolved the capacity to bleach corals but the capacity to take advantage over the other bacteria associated to the host when the necessary conditions are in place. Consequently, in healthy coral reefs, the bacterial community associated to macroalgal surfaces may not represent a threat. Although, macroalgae may act as reservoirs for coral pathogens, there has only been anecdotal reports of bacterial infections in corals attributed to macroalgal contact (e.g., Nugues et al., 2004), and the algal triggered coral disease hypothesis still remains mainly unexplored.

While any bacteria may potentially be adverse to corals, a combination of biotic (e.g., allelopathy) and abiotic (e.g., temperature) factors is regulating microbial composition and abundance on both the coral and the alga (Ritchie, 2006; Mao-Jones et al., 2010; Stratil et al., 2013). Comparably to corals (Ritchie, 2006), algae have the capacity to control the density of specific strains, which coexist in the algal surface biofilm (Barott et al., 2011; Egan et al., 2013). Bacterial regulation may therefore be a key factor preventing bacterial adversity toward corals. *Lobophora* natural products have experimentally demonstrated a broad-spectrum of antibacterial activities (see Vieira et al., 2015 for review). These compounds potentially act as regulators controlling the algal–bacterial communities. Disruption in the coral or algal microbial community equilibrium may result in adverse conditions for corals.

Future studies should be directed at exploring and clearly identifying factors that lead to changes in the microbial community composition.

## Author contributions

CV, AE, OD, and CP designed research; CV, LG, FH, and JG performed the bacterial culture and the *in situ* bioassays; AE, ES, and TA performed the Illumina sequencing analyses; AE, ES, TA, and CV analyzed the Illumina sequencing data; CV and FH analyzed the bioassays data; and CV, AE, TA, LG, ES, FH, CP, and OD co-wrote the paper. All authors critically revisited this work for important intellectual content and approved the final version to be published. All authors agree to be accountable for all aspects of the work.

### Conflict of interest statement

The authors declare that the research was conducted in the absence of any commercial or financial relationships that could be construed as a potential conflict of interest.

## Supplementary material

Table S1 **16S rRNA primers and PCR conditions**.

Table S2 **List of the OTUs identified from *L. rosacea* and *L. monticola***.

Table S3 **The most influential taxon determined with the SIMPER analysis**. The values are the average contributions of each species to the average overall Bray–Curtis dissimilarity.

Table S4 **BLAST-search results for the isolated bacteria strains from *L. rosacea* and *L. monticola***.

Figure S1 **Barplot representation of the relative abundance of phyla associated with the *Lobophora monticola and L. rosacea* characterized by Illumina sequencing in comparison with Barott et al. (2011) results with other macroalgae**.
